# Genome-wide identification of potential biomarkers in multiple myeloma using meta-analysis of mRNA and miRNA expression data

**DOI:** 10.1038/s41598-021-90424-y

**Published:** 2021-05-26

**Authors:** Amit Katiyar, Gurvinder Kaur, Lata Rani, Lingaraja Jena, Harpreet Singh, Lalit Kumar, Atul Sharma, Punit Kaur, Ritu Gupta

**Affiliations:** 1 Bioinformatics Facility, Centralized Core Research Facility, All India Institute of Medical Sciences, Ansari Nagar, New Delhi, 110029 India; 2 ICMR-AIIMS Computational Genomics Centre, Division of Biomedical Informatics, Indian Council of Medical Research, Ansari Nagar, New Delhi, 110029 India; 3 Department of Biophysics, All India Institute of Medical Sciences, Ansari Nagar, New Delhi, 110029 India; 4 Laboratory Oncology Unit, Dr B. R. A. Institute Rotary Cancer Hospital, All India Institute of Medical Sciences, New Delhi, 110029 India; 5 Genomics Facility, Centralized Core Research Facility, All India Institute of Medical Sciences, Ansari Nagar, New Delhi, 110029 India; 6 Department of Medical Oncology, Dr B. R. A. Institute Rotary Cancer Hospital, All India Institute of Medical Sciences, Ansari Nagar, New Delhi, 110029 India

**Keywords:** Cancer, Computational biology and bioinformatics

## Abstract

Multiple myeloma (MM) is a plasma cell malignancy with diverse clinical phenotypes and molecular heterogeneity not completely understood. Differentially expressed genes (DEGs) and miRNAs (DEMs) in MM may influence disease pathogenesis, clinical presentation / drug sensitivities. But these signatures overlap meagrely plausibly due to complexity of myeloma genome, diversity in primary cells studied, molecular technologies/ analytical tools utilized. This warrants further investigations since DEGs/DEMs can impact clinical outcomes and guide personalized therapy. We have conducted genome-wide meta-analysis of DEGs/DEMs in MM versus Normal Plasma Cells (NPCs) and derived unified putative signatures for MM. 100 DEMs and 1,362 DEGs were found deranged between MM and NPCs. Signatures of 37 DEMs (‘Union 37’) and 154 DEGs (‘Union 154’) were deduced that shared 17 DEMs and 22 DEGs with published prognostic signatures, respectively. Two miRs (miR-16–2-3p, 30d-2-3p) correlated with survival outcomes. PPI analysis identified 5 topmost functionally connected hub genes (*UBC, ITGA4, HSP90AB1, VCAM1, VCP*). Transcription factor regulatory networks were determined for five seed DEGs with ≥ 4 biomarker applications (*CDKN1A, CDKN2A, MMP9, IGF1, MKI67*) and three topmost up/ down regulated DEMs (miR-23b, 195, let7b/ miR-20a, 155, 92a). Further studies are warranted to establish and translate prognostic potential of these signatures for MM.

## Introduction

Multiple myeloma (MM) is a neoplasm of plasma cells with heterogeneous clinical symptoms, complex cytogenetic aberrations, multiple copy number variations (CNVs), single nucleotide variations (SNVs), alternate splicing events and epigenetic modifications. Nearly 50% of the MM patients have hyperdiploid karyotypes^1^ with trisomy of chromosomes 3, 5, 7, 9, 11, 15, 19 or 21 while most of the nonhyperdiploid patients develop recurrent translocations between IgH locus and multiple partner genes (such as t(4;14) involving *MMSET/FGFR3*, t(6;14) *CCND3*, t(11;14) *CCND1*, t(14;16) *MAF* and t(14;20) *MAFB*). The frequently encountered CNVs in MM include gains (1q22, 2p14, 3p24.3, 3q26.2, 5q35.2, 6p24.3, 7q22.1, 8q24.2, 9q34.13, 11q13.2, 12q34.21, 15q24.2, 17q23.2, 19p13.2, 20q11.22, 22q13.1) and losses (1p21.3, 4p15.31, 4q13.1, 6q25.3, 7q11.22, 8p22, 9p24.1, 10q24.33, 12p13.1, 12q21.33, 13q21.33, 14q32.32, 16p13.3)^1,2^. In addition, a number of driver mutations occur in genes such as *KRAS, NRAS, FAM46C, BRAF, TP53, MYC* and others that drive disease progression from premalignant Monoclonal Gammaopathy of Undetermined Significance (MGUS) / Smouldering MM (SMM) to active MM^3–5^. Single base substitution based mutation signatures have also been identified in the myeloma genomic landscape that are useful in understanding evolutionary clonal trajectories and other disease aspects in precedence^6^.

Expression profiles of differentially expressed genes (DEGs) are of paramount importance and have provided critical prognostic insights in MM. Recent transcriptome based studies have reported gene expression prognostic (GEP) signatures associated with tumor classification, survival risk prediction^7,8^, progression of MM^7,9–12^, response to drugs^13^, chromosome instbility^14^ and others. The DEGs included in GEP signatures are diverse but closely connected to similar pathways. These genes may relate to kinome^15^, autophagy^16^, cell cycle^10,17^, stemness^18^, cytogenetic abnormalities^9,19^, chromosome 1^20^, homozygous deletions, cell death^21^ and immune^7^ subnetworks. At least 8 to 10 molecular subgroups of MM based on the genomic and transcriptomic patterns have been reported that tend to correlate with different clinical outcomes^8^. Computational and functional analysis of hub genes, nodes, networks and pathways in MM have led to the development of risk scoring systems, relating to the seven genetic subgroups^22^, 70 genes UAMS70 risk signatures^20^, IFM15 risk stratification^17^, 5 gene stemness score^18^, UAMS 17^23^, CINGLEC 214^14^, HOVON-65/GMMG-HD4 EMC 92^9^, HZD 97^21^, M3CN^10^ and others.

However, the prognostic scores derived from GEP signatures have low prediction accuracy and limited power to predict risk or response^23^ perhaps due to MM heterogeneity and complex interactions between malignant plasma cells and bone marrow environment. A landmark study reported GEP prognostication could be improved when a combination of EMC92 + HZDCD^24^ was used. A similar integrative M3CN network study^10^ on MMRF-CoMMpass cohort unified eight prognostic gene signatures and demonstrated significantly improved prognostic performance.

Alterations in expression profiles of genes and small non-coding RNAs, especially, the miRNA, are frequently encountered in MM. Global miRNA expression studies^25–28^ have elucidated a multitude of DEMs in MM. DEMs have been associated with pathogenesis of MM, drug resistance, clinical presentation of disease and clinical outcomes^29–34^. For instance, IL6 inducible miR-21 has been observed at higher expression levels in MM than normal PCs (NPCs)^33^. Similarly, miR-106b, miR-181a, miR-181b, miR-1, miR-133a are upregulated in MGUS while miR-17, miR-32 are upregulated exclusively in MM^33^. Another study^30^ reported decreased levels of let-7a, let-7b, miR-15a, miR-16, miR-20a, and miR-106b both in bone marrow and blood plasma of MM as compared to controls. Aberrant levels of let-7i, miR-15a, miR-16 and miR-106b were found in serum of MGUS while miR-21, miR-223 and miR-361 were deranged exclusively in MM, indicating their roles in early and later events in progression respectively^30^. Correlations of miRs with drug resistance such as miR-29b, miR-202, miR-451 with Bortezomib, miR-125b, miR-137 with Dexamethasone and; miR-140, miR-451 and miR- 152 with Melphalan have been reported^35,36^. Some of the DEMs occur in association with specific cytogenetic subgroups of MM^37^. For example, 1q gain has been correlated with overexpression of miR-1231, 205, 215, 488; 19q gain with upregulation of miR-520a-5p, miR-518d-5p, miR-498, miR-520 g; del13q with downregulated miR15a/16 cluster, miR-17–92 family (miR-17, miR-19a, miR-20a); and 17pdel with reduced expression of miR-22. Similarly, deregulation of miR-133b, miR-135b, miR-155,miR-193a, miR-203, miR-146a, miR-215, miR-342, miR-375, miR-650 have been correlated with t(4;14), miR-95, miR-125a, miR-184, miR-199a, miR-215, miR-375, miR-650, miR-99 with t(11;14), and miR-1, miR-99b, miR-125a, miR-133a, miR-135b, miR-196b, miR-214, miR-375, miR-642 with t(14;16)^32–34,38,39^. In addition, aberrant miRs have been associated with inferior (miR-19a, miR-16, miR-19b) or superior survival (miR-194, miR-153, miR-455) outcomes in MM^35^. Recent studies have established the prognostic, predictive and diagnostic potential of not only cellular but also circulating miRNAs in plasma and other body fluids in MM^40^.

Even though a series of MM associated potential DEM/DEG signatures have been identified across several studies over the years, these remain mostly heterogeneous and challenging to interpret in clinics. There are still unresolved questions such as their mutual interdependencies, interactions with microenvironment and their combinatorial synergistic prognostic and therapeutic potentials. There are still lacunae in our knowledge and more studies are needed to understand the signatures that are best valued in clinics for fast and early prognostication of newly diagnosed MM patients. It is thus postulated that a comprehensive analysis of individual GEP identifiers in MM PCs as compared to normal PCs across multiple studies will help unfold common signatures with potential prognostic significance. In this regard, we have performed a meta-analysis of available multiple datasets of DEGs and DEMs in MM patients to derive a unified set of core GEP signatures. We have identified a combination of ‘Union 154’ DEGs and ‘Union 37’ DEMs that may aid in achieving improved prognosis and clinical applicability.

## Methods

### Inclusion and exclusion criteria for published datasets

The keyword “multiple myeloma” with “homo sapiens” was used to mine the publicly available datasets from the Gene Expression Omnibus (GEO) database^41,42^ of NCBI (http://www.ncbi.nlm.nih.gov/geo/) for miRNA and mRNA expression profiles found in Multiple Myeloma (MM) patients and healthy controls. Datasets obtained from Monoclonal Gammopathy of Undetermined Significance (MGUS), Smoldering Multiple Myeloma (SMM) and Plasma Cell Leukemia (PCL) patients were excluded since the data size was limited. Data emerging from cells or cell lines that were cultured in vitro and/or treated with drugs too were omitted in this study.

### miRNA and mRNA expression datasets

Expression profiles from nine publicly available datasets of mRNAs (GEO accession: GSE125361, GSE13591, GSE16558 and GSE39754), and miRNAs (GEO accession: GSE125363, GSE16558, GSE17306, GSE17498, GSE24371 and GSE49261) associated with MM were retrieved from publicly available GEO repository (Table S1 in Supplementary File 1) as per the inclusion and exclusion criteria. Among these, two datasets of miRNA and mRNA expression profiles (44 newly diagnosed MM patients and 4 controls) were generated in-house by Agilent arrays. These miRNA and mRNA datasets have been submitted to GEO and assigned with accession numbers GSE125363 and GSE125361, respectively. In this study, miRNA expression profiles corresponding to 247 MM and 31 healthy control plasma cell samples in total, whereas mRNA expression profiles representing 407 MM and 20 healthy plasma cell samples in total were collated and analyzed (Table S1 in Supplementary File 1). Datasets obtained from GEO repository were not subjected to any additional normalization, as all the data obtained had already been processed/ normalized and were cross-comparable.

### Preprocessing and mining of DEMs/DEGs from GEO repository

GEO2R^42^ (http://www.ncbi.nlm.nih.gov/geo/geo2r/) web tool was used to identify DEGs and DEMs among MM and control plasma cell samples. GEO2R compared two or more groups of samples in a GEO profile using the GEOquery and Limma (Linear Models for Microarray Analysis) R package^43^. Limma used linear model statistics to find genes that were differentially expressed between the patient and control groups. The t-test and the Benjamini and Hochberg method were used to calculate the p-values and false discovery rate (FDR), respectively^44^. The adjusted (adj.) *p* ≤ 0.05 and |logFC|≥ 1.5 were set as the cut-off criterion for identifying DEGs and DEMs.

### Genome-wide miRNA and mRNA expression profiling

Total RNA was isolated from CD138 + plasma cells enriched with MACS beads (Miltenyi Biotech, Germany), collected from 44 newly diagnosed treatment naïve MM patients diagnosed as per IMWG guidelines^45^ (Table S2 in Supplementary File 1) and 4 controls (pooled from 10 Hodgkin's disease bone marrow samples). Total RNA was extracted using the miRVana miRNA isolation kit (Thermofisher Scientific, MA, USA).

For the genome wide miRNA expression profiling, RNA was labeled and hybridized to an unrestricted human microRNA v19 Microarray slide (Agilent 046,064, GPL18044) (Agilent Technologies, Santa Clara, CA, USA) according to the manufacturer's protocol. Briefly, 100 ng of total RNA was labeled with Cyanine3 (Cy3) using miRNA Complete Labeling and Hybridization Kit (Agilent Technologies, Santa Clara, CA, USA). The Cy3-labeled samples were resuspended in hybridization buffer and hybridized onto Human miRNA 8X60K format microarrays (Agilent Technologies, Santa Clara, CA, USA) at 55 °C for 20 h. After hybridization, microarrays were washed with gene expression wash buffer and the fluorescent signals were scanned using SureScan microarray scanner D (Agilent Technologies, Santa Clara, CA, USA) using one colour scan settings (Scan resolution 3 μm, Dye channel set to Green, Green PMT = 100%). The data generated on miRNA expression in MM using microarrays has been submitted to GEO database with accession no GSE125363.

To correlate whether the miRNA alteration affects gene expression, mRNA expression array analysis was also performed on 44 MM patient samples and 4 controls (pooled from 10 Hodgkin's disease bone marrow samples). Double-stranded cDNA was generated from 200 ng total RNA (isolated with miRVana kit) using the low input quick amp labelling kit (Agilent Technologies, Agilent Technologies, Santa Clara, CA, USA) using T7 primer, dNTPs and affinity script RNase block. Next, cDNA was transcribed to cRNA using T7 RNA polymerase and NTP mix and labeled with Cyanine3 using Cy3-CTP. The labeled cRNA was purified according to manufacturer’s protocol using RNAeasy extraction kit (Qiagen, Hilden, Germany). The concentration of Cyanine3 and cRNA was measured using NanoDrop ND1000 spectrophotometer. Samples with specific activity ≥ 6 pmol Cy3/µg cRNA were hybridized onto a SurePrint G3 human GE v3 8 × 60 K microarray slide (Agilent 072,363, GPL20844) (Agilent technologies, Santa Clara, CA, USA), and incubated for 17 h at 65 °C in a hybridization oven. The slides were washed and scanned in SureScan microarray scanner D (Agilent technologies, Santa Clara, CA, USA) with scan settings (Scan resolution 3 μm, Dye channel set to Green, Green PMT = 100%). The data generated on mRNA expression in MM has been submitted to GEO database with accession no. GSE125361.

### Preprocessing and mining of DEMs/DEGs from Agilent array

Microarray images (*.tiff) obtained from SureScan scanner were quantified using Agilent Feature Extraction Software (version 11.5.1.1) (Agilent Technologies, Santa Clara, CA, USA). The raw data obtained (tab-delimited text file per hybridisation) was subsequently processed with the Limma R package available in the Bioconductor repository (http://www.bioconductor.org). Limma used linear model statistics to find genes that were differentially expressed between the patients and controls. The raw intensity data were background corrected using normexp method and subsequently normalized using quantile method for one-color. Expression level variations between replicates were analyzed by pairwise comparisons using the lmFit function. The fitted model object was further processed by the eBayes function to produce empirical Bayes test statistics for each gene, including moderated t-statistics, p-values and log-odds of differential expression. The t test and Benjamini and Hochberg method were used to calculate the p-values and false discovery rate (FDR)^44^. The adjusted *p* ≤ 0.05 and |logFC|≥ 1.5 were set as the cut-off criterion for identifying DEGs and DEMs.

### Meta-analysis of DEGs/DEMs datasets

A widely used meta-analysis approach^46–48^ was applied to integrate the gene/miRNA expression profiles obtained independently from GEO repository as well as datasets generated at our centre following microarray hybridization (Table S1 in Supplementary File 1). The gene and miRNA probes were assigned as per HGNC (HUGO Gene Nomenclature Committee) and miRBase-22.1 identifiers, respectively using the g:Profiler^49^ (https://biit.cs.ut.ee/gprofiler/). The differentially expressed genes/miRNAs obtained through R/Bioconductor limma package^43^ from these individual studies were merged by taking the union across them. When multiple probes referred to the same gene/miRNA, the expression values obtained from these probes were minimized to a single value by averaging the expression value (when in the same direction of expression) or were discarded (when had diverse directions of expression). The probes with unknown gene or unknown miRNA identifiers or annotated as antisense RNA, chromosomes, hypothetical loci, non-coding RNAs, non-functional proteins, non-protein coding genes, pseudo-genes and uncharacterized genes were discarded. The DEGs identified were mapped in DisGeNET^50^ to determine their known disease associations.

### miRNA-mRNA target interactions

The target genes of potential DEMs were predicted using miRNet-2.0 (https://www.mirnet.ca/) according to eleven different miRNA databases (TarBase, miRTarBase, miRecords, miRanda, miR2Disease, HMDD, PhenomiR, SM2miR, PharmacomiR, EpimiR and starBase). The miRNA-mRNA pairs with inverse correlation expression trends were filtered for downstream analysis.

### Core analysis using IPA

Ingenuity Pathway Analysis (IPA, Ingenuity Systems, USA; www.qiagen.com/ingenuity) was used to identify the biological functions, diseases, canonical pathways, and regulatory networks of the functional miRNA-mRNA target interactions. Tab-delimited text files containing gene/miRNA IDs, expression data (fold change), and *p*-values were uploaded into IPA for their core analysis. The statistical significance of the enrichment was calculated using hypergeometric test and adjusted by FDR method (adj. *p*-value ≤ 0.05). The top functions (molecular, cellular and biological), diseases, toxicology, and gene signaling networks were calculated using IPA-generated negative logarithm p-values i.e., -log10(p-value) and associated Z- and network scores.

### Construction of protein–protein interaction (PPI) network

To examine the interactive associations among the DEGs at the protein level, MM related genes were mapped on protein–protein interaction (PPI) data using NetworkAnalyst^51^ (version 3.0; http://www.networkanalyst.ca). The network was built based on the original seed proteins through executing the minimum interaction network by trimming the first-order network to keep only those nodes that are necessary to connect the seed nodes. Literature-curated comprehensive PPI data was used to predict interaction network^52^. Network modules containing densely connected group of proteins were predicted using the random walk approach. The significant p-value of a given module was calculated with Wilcoxon rank-sum test^53^. The enriched pathways of DEGs in significant modules (≥ 10 DEGs) were analysed with a threshold of *p* ≤ 0.05 using DAVID (database for annotation, visualization and integrated discovery) functional annotation tool.

#### Biomarker candidates and TF regulatory network

The candidate gene biomarkers were predicted using Ingenuity Pathway Analysis (IPA-biomarkers analysis; http://www.ingenuity.com). The adjusted (adj.) *p* ≤ 0.05 and |logFC|≥ 1.5 were set as the cut-off criterion. Upstream regulators (TFs) of biomarker candidates were predicted using NetworkAnalyst (http://www.networkanalyst.ca). TF-gene interaction analysis was performed using the ENCODE database. The miRNA-disease association was predicted by HMDD v3.2 (Human microRNA Disease Database; http://www.cuilab.cn/hmdd) and miRNet 2.0 (https://www.mirnet.ca/). The regulatory associations between TFs and miRNAs were predicted using TransmiR v2.0 (http://www.cuilab.cn/transmir). Functional and pathway enrichment analyses of upstream regulators were investigated using DAVID (https://david.ncifcrf.gov/) with adjusted (adj.) p ≤ 0.05 and |logFC|≥ 1.5 cut-off criteria.

#### Survival analysis

Sigmaplot 14.0 was used to estimate Kaplan–Meier plots for overall survival (OS) and progression free survival (PFS). The survival analysis was carried out on 35 patients in whom clinical data was available. Comparisons between DEMs were analyzed using means of log-rank test and *p* ≤ 0.05 as cut-off for statistical significance.

### Ethical clearance

The study on 44 MM patients collected from the outpatient department of the All India Institute of Medical Sciences (AIIMS), New Delhi was conducted in compliance with ethical guidelines of the AIIMS and after obtaining approval from the AIIMS ethics committee. Study individuals were enrolled following their voluntary written informed consent.

## Results

### Identification of DEGs/DEMs in MM compared with NPCs

Following consolidation of data and its meta-analysis, a total of 100 DEMs and 1,362 DEGs were identified between multiple myeloma (MM) and normal plasma cells (NPC) with FDR ≤ 0.05 and logFC ≥ 1.5 threshold values (Supplementary Tables S3-S4). Among these DEMs, 43 miRNAs were upregulated, and 57 miRNAs were downregulated, whereas 708 DEGs were upregulated and 654 genes were downregulated in MM (Tables S3-S4 in Supplementary File 1). The DEGs identified from this study were mapped across the available data of MM present in DisGeNET which established their known associations with MM (Table S5 in Supplementary File 1).

### MicroRNA–mRNA regulatory network analysis

**Figure 1:**
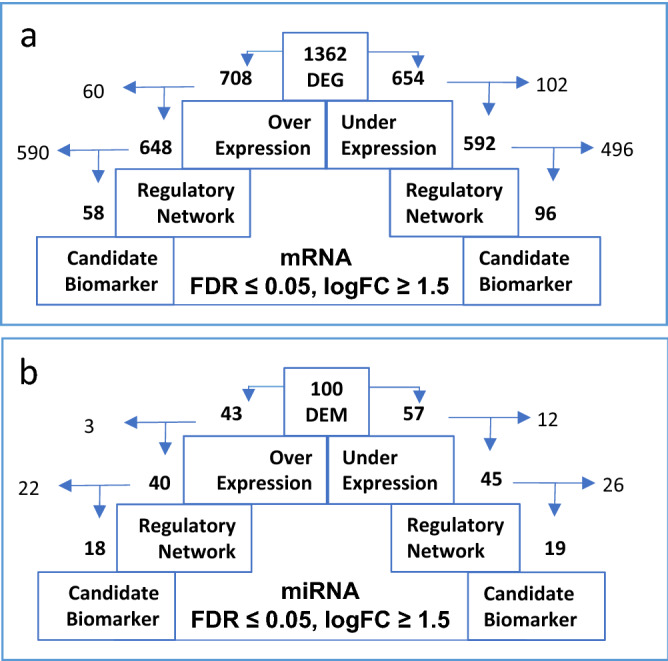
Analysis flow of (**a**) DEGs and (**b**) DEMs in MM showing number of genes/ miRNAs with upregulated expression on left hand side and downregulated expression on the right side. (**a**) Out of 1,362 DEGs, 708 were over-expressed, out of which 648 were found to be involved in regulatory network with miRNA and 58 DEGs ultimately showed up as possible candidate biomarker in MM. Similarly, 96 of downregulated DEGs were found to have the possible potential to be investigated further as candidate biomarkers. (**b**) Expression of 45 and 40 out of 100 DEMs was downregulated or upregulated respectively, and ultimately 19 and 18 subsets of these turned up to be possible candidate biomarkers for MM.

To verify the targets of differentially expressed miRNAs in MM datasets, miRNA–mRNA regulatory network was constructed using the 100 DEMs and 1,362 DEGs. The analysis showed an association of 85 DEMs, including 40 upregulated and 45 downregulated miRNAs, with 1,240 target genes, including 592 downregulated and 648 upregulated DEGs in MM (Fig. 1). The observed targets were statistically significant with p-value ≤ 0.05 and fold change ≥ 1.5. The most significantly upregulated and downregulated miRNAs in MM were observed to be hsa-miR-191-5p (4.80 logFC) and hsa-miR-155-5p (− 4.69 logFC), respectively, whereas the most significantly upregulated and downregulated DEGs were *TNFRSF17* (4.91 logFC) and *DEFB1* (− 4.56 logFC), respectively (Table 1; Table S6 and S7 in Supplementary File 1). hsa-miR-155-5p (-4.69 logFC) was predicted to target maximum number of genes (315 upregulated DEGs), whereas hsa-miR-602 (2.20 logFC) was predicted to target the least number of genes (2 downregulated DEGs) (Table S8 in Supplementary File 1). Conversely, several genes were predicted to be the common targets of different miRNAs (Table S9 in Supplementary File 1).

**Table 1:** List of top 5 up- and down-regulated miRNAs and genes in multiple myeloma.

Accession no	miRNA name (miRBase-22.1)	adj.P.Val	logFC	Regulation
MIMAT0000440	hsa-miR-191-5p	1.72E-03	4.80	Up
MIMAT0000420	hsa-miR-30b-5p	1.27E-02	4.78	Up
MIMAT0003326	hsa-miR-663a	3.07E-04	4.58	Up
MIMAT0000243	hsa-miR-148a-3p	5.66E-13	4.38	Up
MIMAT0000433	hsa-miR-142-5p	3.07E-04	4.20	Up
MIMAT0000646	hsa-miR-155-5p	2.97E-03	-4.69	Down
MIMAT0000753	hsa-miR-342-3p	1.57E-05	-3.74	Down
MIMAT0000085	hsa-miR-28-5p	4.57E-09	-3.13	Down
MIMAT0003320	hsa-miR-650	2.97E-02	-3.10	Down
MIMAT0000266	hsa-miR-205-5p	3.29E-09	-3.07	Down

Accession no	Gene name	adj.P.Val	logFC	Regulation
HGNC:11,913	TNFRSF17	0.00E + 00	4.91	Up
HGNC:13,310	GPRC5D	0.00E + 00	4.49	Up
HGNC:2318	CPNE5	0.00E + 00	4.29	Up
HGNC:21,063	MOXD1	2.00E-02	4.02	Up
HGNC: 17,825	PLA2G16	0.00E + 00	3.99	Up
HGNC:2766	DEFB1	2.00E-02	-4.56	Down
HGNC:1990	CKAP2	0.00E + 00	-4.00	Down
HGNC:11,763	TFRC	2.00E-02	-3.95	Down
HGNC:26,260	TMEM156	7.65E-04	-3.93	Down
HGNC:1036	BEX1	1.00E-02	-3.75	Down

### Correlation between canonical pathways, diseases and functions

**Figure 2:**
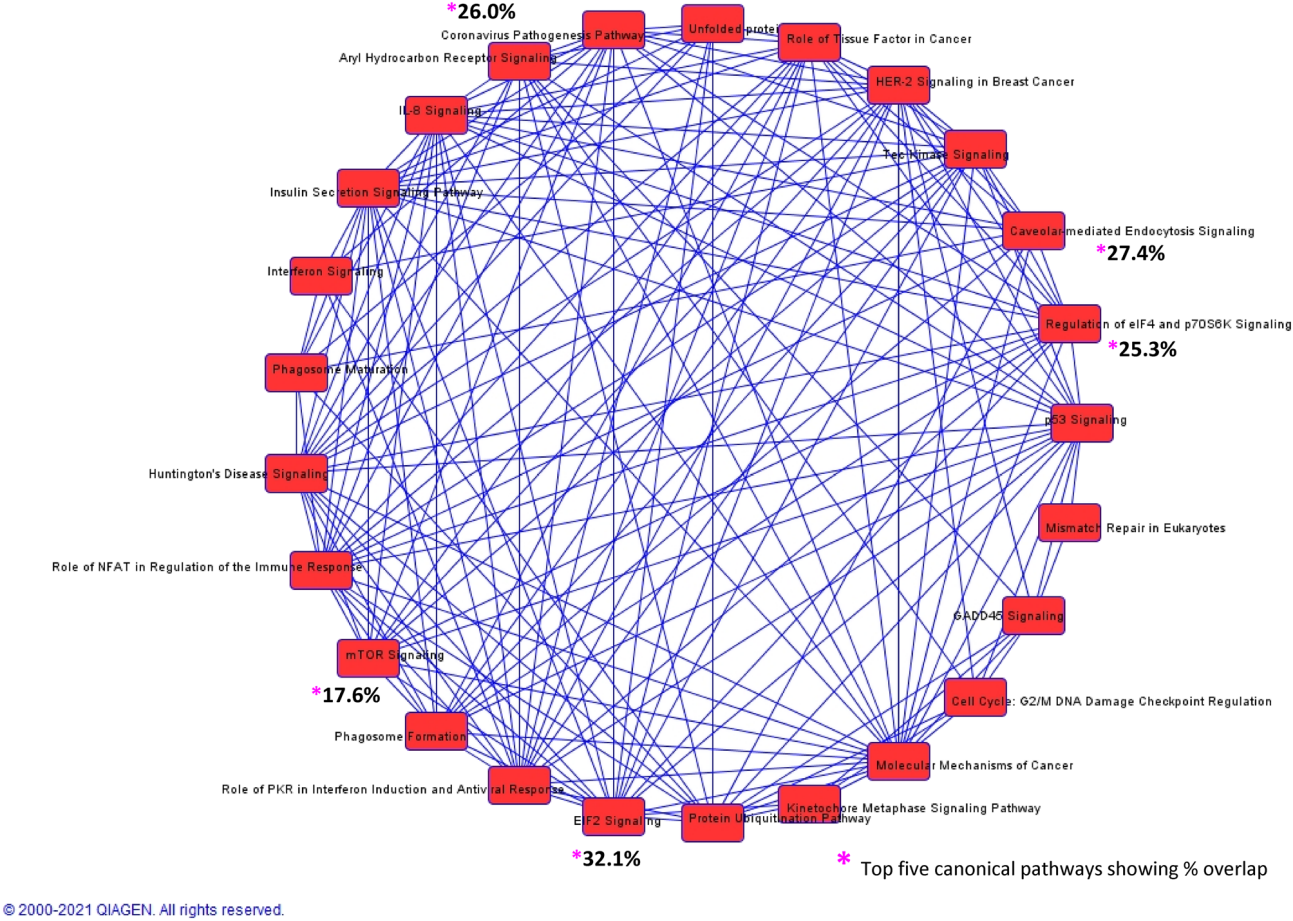
Overlapping canonical pathways generated by IPA (QIAGEN IPA; http://www.ingenuity.com). The figure shows overlapping canonical pathways associated with differential proteins. The nodes represent pathways and edges are labeled with the number of common proteins connecting each node. Top 5 significant pathways are marked with * followed by % overlap across pathways.

To gain further insights into the pathogenesis of MM, all significant MM-correlated genes/miRNAs were investigated by IPA core analysis that revealed 555 human canonical pathways significantly enriched for 698 overlapping genes associated with MM (Table S10 in Supplementary File 1).The top five significant enriched pathways based on their significance (lowest BH-adjusted *p*-value ≤ 0.05) were EIF2 signaling (9.90E-34), regulation of eIF4 and p70S6K signaling (5.62E-16), coronavirus pathogenesis pathway (2.31E-15), mTOR signaling (2.49E-09), and caveolar-mediated endocytosis signaling (5.46E-09) (Fig. 2).

DEGs and DEMs were further investigated for their involvement in most enriched diseases and for their functions in multiple myeloma. On annotation, most of the DEGs were found to be involved in cancer, organismal injury and abnormalities, immunological disease, connective tissue disorder, inflammatory disease (Figure S1a in Supplementary File 2), whereas DEMs were found to be enriched in cancer, organismal injury and abnormalities, reproductive system disease, inflammatory disease, and inflammatory response (Figure S1b in Supplementary File 2). The topmost significant diseases and biofunctions identified for DEMs and DEGs are shown in Table 2. Besides the leading pathways and cellular functions, gene networks were constructed to connect key genes and enriched categories of diseases and functions based on the correlation between DEGs. Core analysis-based network revealed 25 significant networks and each individual network had a maximum of 35 focus genes. Top ranked network (network 1) with an IPA score of 49 contained 35 focus molecules. Top functions of genes associated with network 1 were mainly connected to cellular assembly and organization, energy production, nucleic acid metabolism. Likewise, miR-network consisted of 8 major networks with a maximum of 24 focus genes. Most of the genes in miRNA network 1 were mainly connected to cancer, organismal injury and abnormalities and reproductive system disease. Gene/miRNA networks and their related top diseases and functions are listed in Table 3 and Table S11 in Supplementary File 1.

**Table 2:** Top five diseases and bio functions identified by IPA analysis of miRNA and mRNA in MM.

Name (miRNA)	*p*-value range	Focus molecules
*Diseases and Disorders*		
Cancer	4.96E-02—3.57E-30	52
Organismal Injury and Abnormalities	4.96E-02—3.57E-30	60
Reproductive System Disease	1.62E-02—3.57E-30	42
Inflammatory Disease	4.00E-02—2.49E-25	39
Inflammatory Response	4.00E-02—2.49E-25	33
*Molecular and Cellular Functions*		
Cellular Development	4.96E-02—3.34E-14	42
Cellular Growth and Proliferation	4.96E-02—3.34E-14	39
Cellular Movement	4.52E-02—3.96E-08	24
Cell Cycle	4.52E-02—3.48E-07	10
Cell Death and Survival	4.11E-02—6.36E-06	24
*Physiological System Development and Function*		
Organismal Development	4.96E-02—3.06E-11	19
Digestive System Development and Function	1.02E-08—1.02E-0	6
Hepatic System Development and Function	1.02E-08—1.02E-0	6
Organ Development	1.02E-08—1.02E-0	6
Cardiovascular System Development and Function	4.96E-02—2.94E-07	14

Name (genes)	*p*-value range	Focus molecules
*Diseases and Disorders*		
Cancer	1.70E-09—1.90E-62	1324
Organismal Injury and Abnormalities	1.70E-09—1.90E-62	1339
Immunological Disease	1.04E-09—1.45E-39	630
Connective Tissue Disorders	1.03E-10—2.35E-35	258
Inflammatory Disease	1.42E-09—2.35E-35	337
*Molecular and Cellular Functions*		
Cell Death and Survival	1.70E-09—1.24E-47	594
Protein Synthesis	6.71E-13—6.21E-41	257
RNA Damage and Repair	9.02E-35—2.44E-35	54
Cellular Compromise	3.75E-18—2.72E-33	189
Cellular Development	8.50E-10—3.16E-27	490
*Physiological System Development and Function*		
Organismal Survival	3.14E-22—6.61E-28	411
Immune Cell Trafficking	1.54E-09—1.81E-26	259
Lymphoid Tissue Structure and Development	1.24E-09—3.59E-26	273
Hematological System Development and Function	1.54E-09—1.10E-25	403
Tissue Morphology	1.03E-09—1.37E-22	315

**Table 3:** Top five associated network functions predicted by an IPA analysis of miRNA and mRNA in MM.

ID	Associated Network Functions (miRNAs)	Score	Focus Molecules
1	Cancer, Organismal Injury and Abnormalities, Reproductive System Disease	58	24
2	Glomerular Injury, Inflammatory Disease, Inflammatory Response	43	19
3	Neurological Disease, Organismal Injury and Abnormalities, Psychological Disorders	32	15
4	Digestive System Development and Function, Gastrointestinal Disease, Hepatic System Development and Function	19	10
5	Glomerular Injury, Inflammatory Disease, Inflammatory Response	2	1
ID	Associated Network Functions (genes)		
1	Cellular Assembly and Organization, Energy Production, Nucleic Acid Metabolism	49	35
2	RNA Post-Transcriptional Modification, Nucleic Acid Metabolism, Small Molecule Biochemistry	46	34
3	Cell Cycle, Cellular Assembly and Organization, DNA Replication, Recombination, and Repair	43	33
4	Drug Metabolism, Small Molecule Biochemistry, Cellular Compromise	41	32
5	Infectious Diseases, Post-Translational Modification, Developmental Disorder	38	31

### Identification of functional modules in PPI network

Protein–protein interactions (PPI) network was constructed using aberrantly expressed genes identified in MM to predict biologically significant modules containing a group of proteins that execute similar functions. The minimum interaction network scattered in 1–3 sub-networks including one big network with highest nodes and edges. The network analysis disclosed 1,136 seeds (91.61% of DEGs) associated with 1,937 nodes in the network. The modules containing a group of proteins with identical functions were detected using the random walk approach. A total of 22 significant independent functional modules were observed, whereas 13 modules (module no: 0, 1, 2, 3, 4, 5, 6, 7, 9, 11, 12, 13, and 16) were highly connected with more than 10 nodes and *p* ≤ 0.05 (Table 4; Table S12 in Supplementary File 1). Out of 1,136 seed nodes, a total of 34.68% (n = 394) nodes were observed with ≥ 10 degrees or connections with other nodes (Table S12 in Supplementary File 1). The betweenness centrality of nodes ranged between of 13.37 to 741,529.3 in the constructed network. All 394 nodes were observed to be targeted by at least one MM-associated DEMs (Table S13 in Supplementary File 1).

**Table 4:** Top five significant functional modules and associated hub genes.

^#^Module	Size	*P*-value	Gene Id	Gene Symbol	*Degree	Betweenness
0	402	3.64E-11	672	BRCA1	51	12,309.77
			1026	CDKN1A	41	9837.73
			5111	PCNA	37	5809.36
			983	CDK1	30	4144.93
			5347	PLK1	26	5018.87
			4176	MCM7	24	2771.13
			991	CDC20	23	1982.08
			472	ATM	22	2491.62
			890	CCNA2	21	1781.56
			1029	CDKN2A	20	3589.92
1	373	4.57E-08	7316	UBC	358	46,319.96
			3676	ITGA4	97	1251.95
			7412	VCAM1	95	1200.74
			7415	VCP	90	1340.9
			3312	HSPA8	88	987.02
			3326	HSP90AB1	83	1125.47
			3309	HSPA5	81	795.28
			8452	CUL3	67	541.42
			3303	HSPA1A	66	565.57
			203,068	TUBB	66	528.71
2	238	1.99E-02	3725	JUN	52	4409.1
			6772	STAT1	47	4410.76
			1958	EGR1	44	4338.56
			5925	RB1	39	3345.44
			3659	IRF1	34	1100.71
			7421	VDR	29	2058.43
			5966	REL	26	1825.76
			6667	SP1	25	2321.16
			7157	TP53	23	1708.21
			2033	EP300	23	929.11
3	58	3.34E-02	6194	RPS6	51	42.61
			6191	RPS4X	51	37.5
			6210	RPS15A	51	33.01
			6207	RPS13	50	38.73
			6189	RPS3A	49	52.8
			6228	RPS23	49	32.17
			6188	RPS3	48	26.02
			6202	RPS8	48	20.13
			6217	RPS16	48	18.54
			6129	RPL7	47	16.68
4	45	3.26E-02	1994	ELAVL1	33	870
			351	APP	11	250

^#^Top 5 modules based on size

*Top 10 hub genes based on degree.

The top five highly connected hub nodes included *UBC, ITGA4, HSP90AB1, VCAM1* and *VCP* (Table S12 in Supplementary File 1). Module-wise distribution of top three highly connected hub nodes encompassed *BRCA1, CDKN1A* and *PCNA* in module 0 (Figure S2a), *UBC, ITGA4* and *VCAM1* in module 1 (Figure S2b) and *JUN, STAT1* and *EGR1* in module 2 (Figure S2c in Supplementary File 2).

### Identification of biomarker candidates for multiple myeloma

**Figure 3:**
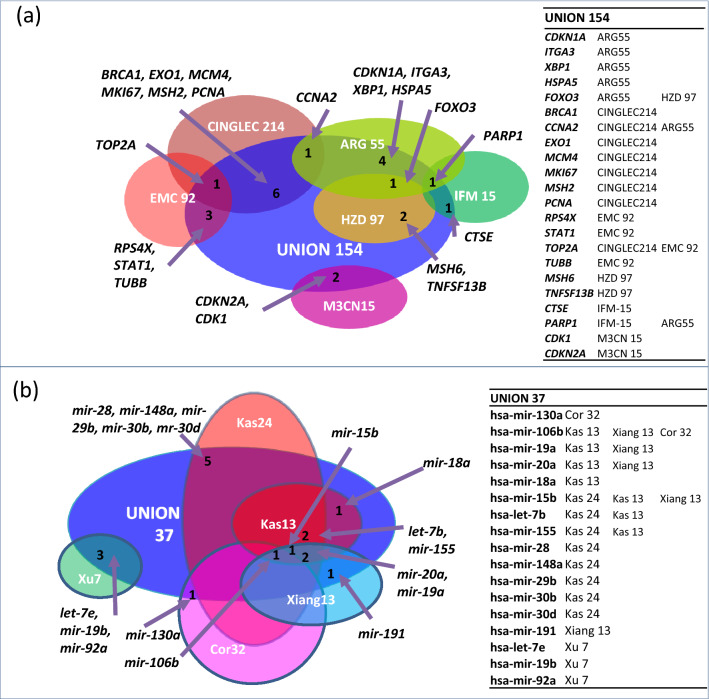
A comparison of commonality between (**a**) Union 154 DEG and (**b**) Union 37 DEM signatures found in this study with analogous published signatures showing an overlap of 22 DEGs and 17 DEMs, respectively^9,10,14,16,17,21,25,26,28,54^.

The common molecular biomarker candidates among DEGs and DEMs for diagnosis, disease progression, efficacy, prognosis, response to therapy and safety were identified using the IPA software and HMDD/miRNet database, respectively (Table 5). The analysis revealed 154 (12.42%) potential biomarkers out of 1,240 observed DEGs that could bear clinical value for MM and were designated as ‘Union 154’ signature (Fig. 3a). These included common biomarker candidates predominantly with diagnosis (n = 82; 63.25%), efficacy (n = 90; 58.44%), prognosis (n = 56; 36.36%), disease progression (n = 21; 13.64%), response to therapy (n = 23; 14.94%), and safety (n = 9; 5.84%) (Tables S14 and S15 in Supplementary File 1). Among the target gene candidate biomarkers, 42.21% (n = 65) of targets qualified for more than one role (Supplementary Tables S14 and S15). For example, gene CDKN2A was observed to be implicated in six biomarker applications including diagnosis, disease progression, efficacy, prognosis, response to therapy and safety.

**Table 5:** Common molecular biomarker candidates for diagnosis, disease progression, efficacy, prognosis, response to therapy and safety in multiple myeloma.

*Gene Biomarker	Description	Regulation	Biomarker application(s)
CDKN2A	Cyclin dependent kinase inhibitor 2A	Up	Diagnosis,disease progression,efficacy,prognosis,response to therapy,safety
HGF	Hepatocyte growth factor	Up	Diagnosis,disease progression,efficacy,prognosis
IGF1	Insulin like growth factor 1	Up	Diagnosis,efficacy,prognosis,safety
CDKN1A	Cyclin dependent kinase inhibitor 1A	Up	Diagnosis,efficacy,prognosis,response to therapy
STAT1	Signal transducer and activator of transcription 1	Up	Diagnosis,efficacy,prognosis,response to therapy
MKI67	Marker of proliferation Ki-67	Down	Diagnosis,disease progression,efficacy,prognosis,response to therapy
PTK2	Protein tyrosine kinase 2	Down	Diagnosis,disease progression,efficacy,prognosis
MMP9	Matrix metallopeptidase 9	Down	Diagnosis,disease progression,efficacy,prognosis
TOP2A	DNA topoisomerase II alpha	Down	Diagnosis,efficacy,prognosis,response to therapy
VCAM1	Vascular cell adhesion molecule 1	Down	Diagnosis,disease progression,efficacy,prognosis

^**★**^ Gene candidates with ≥ 4 biomarker applications.

In addition, miRNA disease databases such as HMDD and miRNet revealed 37 aberrantly expressed miRNAs as potential biomarkers with clinical utility for MM (Table S16 in Supplementary File 1) and were designated as ‘Union 37’ signature (Fig. 3b). A systematic literature review of ‘Union 37’ signatures disclosed that 29.73% (n = 11) miRNAs were known circulating biomarkers for diagnostics and prognostics in MM. Some of these miRNAs were identified as epigenetically regulated miRNAs (n = 4), as therapeutic targets (n = 7) and dysregulated miRNAs that resulted in MM disease phenotype (n = 7) and are given in Table 6.

**Table 6:** List of miRNAs that could result in disease phenotypes (multiple myeloma) when permutated.

miRNA biomarker	Regulation	Evidence	Description	Causality
hsa-mir-148a	Up	Target gene	MiR-148a participates in the growth of RPMI8226 multiple myeloma cells by regulating CDKN1B	YES
hsa-mir-23b	Up	Transcription factor target	miR-23b/SP1/c-myc forms a feed-forward loop supporting multiple myeloma cell growth	YES
hsa-mir-29a	Up	Genetics_overexpression_suppress	In addition, ectopic expression of miRNA-29a or exposure to PRIMA-1Met reduced cell proliferation and induced apoptosis in MM cells	YES
hsa-mir-29b	Up	Therapeutic target	miR-29b-based epi-therapeutic approaches in the treatment of this malignancy	YES
hsa-mir-29b-1	Up	Target gene	Overexpression of microRNA-29b induces apoptosis of multiple myeloma cells through down regulating Mcl-1	YES
hsa-mir-16–1	Down	Genetics_knock down_promote	The common loss of miR-15a and miR-16–1 in CLL, as well as the loss of 13q14 in mantle cell lymphoma (50 percent of cases), multiple myeloma (16 to 40 percent) and prostate cancer (60 percent), strongly suggests that these two miRNAs act as tumor suppressor genes. While their full target complement is unknown, they appear to mediate their effects largely by down-regulating the anti-apoptotic protein BCL2. This protein is often found expressed at high levels in CLL and is thought to be important for the survival of the malignant cells	YES
hsa-mir-16–1	Down	Target gene	miR-15a and miR-16 affect the angiogenesis of multiple myeloma by targeting VEGF	YES
hsa-mir-19a	Down	Genetics_overexpression_promote	miR-19a is overexpressed significantly in Lp-1 and U266 multiple myeloma cells, and promots the proliferation and invasion of the myeloma cells, but inhibits their apoptosis	YES
hsa-mir-20a	Down	Target gene	Effects of microRNA-20a on the proliferation, migration and apoptosis of multiple myeloma via the PTEN/PI3K/AKT signaling pathway	YES

### Effect of DEGs/DEMs on clinical outcomes

**Figure 4:**
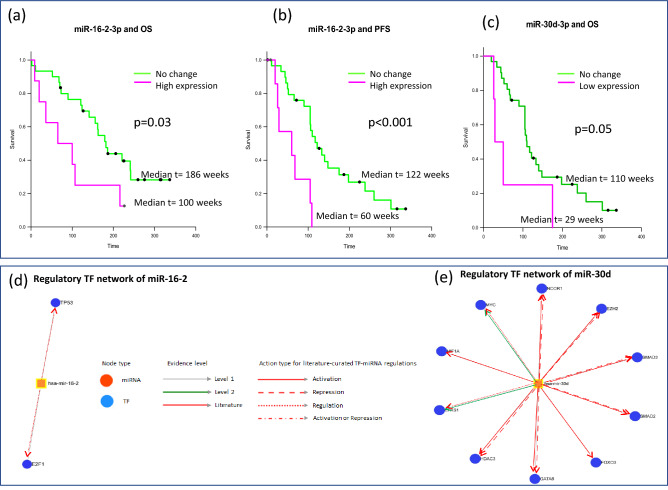
Kaplan Meier plots showing associations of (**a**) miR-16–2-p with OS, (**b**) miR-16–2-3p with PFS and (**c**) miR-30d-3p with OS. Regulatory transcription factor networks of miR-16–2 and of miR-30d are shown in (**d**) and (**e**) respectively.

A significant correlation of differential expression of miR-30d-3p with PFS (*p* = 0.05) and of miR-16–2-3p with OS (*p* = 0.03) and PFS (*p* < 0.001) was observed (Fig. 4a–c). The miR-16–2-3p interacted with two predominant transcription factors (P53, E2F1) (Fig. 4d) while miR-30d-3p interacted with multiple transcription factors (EPAS1, EZH2, FOXO3, GATA6, HDAC3, HIF1A, MYC, NCOR1, SMAD2, SMAD3) (Fig. 4e) (Table S17 in Supplementary file 1).

### TF-gene/miRNA coregulatory networks

**Figure 5:**
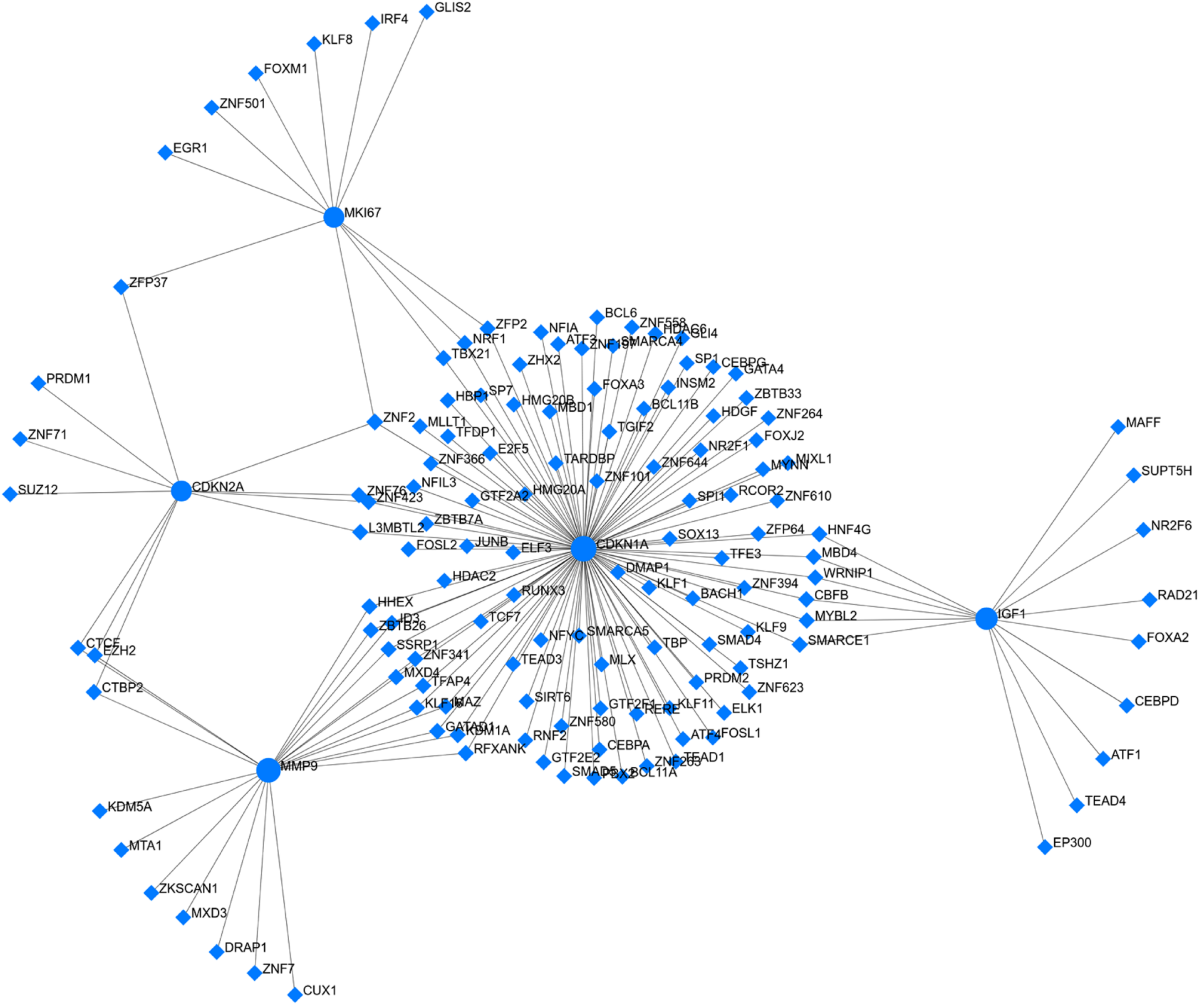
TF-gene biomarker regulatory network generated by NetworkAnalyst (version 3.0; http://www.networkanalyst.ca). The network revealed 164 interaction pairs among 5 seed genes and 139 transcription factors (TFs). Blue circle stands for the seed gene and blue diamond stands for the transcription factor.

We further investigated the TF-miRNA-target gene regulatory network for meta-signature gene/miRNAs identified in this study. The gene-TF regulatory network of 5 gene (≥ 4 biomarkers applications) revealed 164 interaction pairs among 5 seed genes (*CDKN1A, MMP9, CDKN2A, MKI67, and IGF1*) and 139 transcription factors (TFs) (Table S18 in Supplementary File 1). Among them, upregulated gene *CDKN1A* was found to be regulated by 87 TFs, *CDKN2A* was regulated by 12 TFs and *IGF1* was regulated by 10 TFs (Fig. 5). Similarly, the downregulated gene *MMP9* interacts with 20 TFs, and *MKI67* interacts with 10 TFs (Fig. 5). TF-gene interactions are shown in Table S17 in Supplementary File 1.

TF-miRNA regulatory analysis of top 3 up- and downregulated miRNA biomarkers based on the number of targets showed an association with 339 TFs (Table S19 in Supplementary File 1). From the data of TransmiR, downregulated DEMs such as hsa-miR-20a, hsa-mir-155, and hsa-mir-92a were found to be regulated by 143, 114 and 11 TFs, respectively (Figure S3a-c). Likewise upregulated DEMs including hsa-mir-23b, hsa-mir-195 and hsa-let-7b were found to be regulated by 140, 63 and 58 TFs, respectively (Figure S3d-f in Supplementary File 2; Table S17 in Supplementary File 1).

The top 5 enriched biological functions of TFs were investigated and subsequently compared for up-and downregulated meta-signature gene/ miRNAs (Table S20 in Supplementary File 1). TFs of upregulated genes were enriched in pathway namely “transcriptional misregulation in cancer”, whereas TFs of downregulated genes were not observed to be significantly enriched in any pathways. Moreover, we found that the TFs of up- and downregulated miRNAs were commonly enriched in 39 pathways, including MAPK signaling pathway (hsa04010), HIF-1 signaling pathway (hsa04066), cell cycle (hsa04110), wnt signaling pathway (hsa04310), osteoclast differentiation (hsa04380), toll-like receptor signaling pathway (hsa04620), B cell receptor signaling pathway (hsa04662), pathways in cancer (hsa05200), Transcriptional misregulation in cancer (hsa05202), Viral carcinogenesis (hsa05203), microRNAs in cancer (hsa05206), chronic myeloid leukemia (hsa05220), acute myeloid leukemia (hsa05221), small cell lung cancer (hsa05222) and others (Table S20 in Supplementary File 1).

## Discussion

In this study, a meta-analysis of mRNA and miRNA expression profiles has been carried out on more than 600 MM patients including 44 Indian myeloma patients (represented in 9 GSE-GEO datasets) in order to compute altered mRNA and miRNA patterns and potential biomarkers of prognostic clinical relevance in multiple myeloma. Overall, this study has imputed two core signatures, ‘Union 154’ for DEGs and ‘Union 37’ for DEMs in MM that appear to have a unified representation of several other analogous signatures reported in the literature^9,10,14,16,17,21,25,26,28,54^ (Fig. 3a and b).

The present study has revealed that 85% (85/100) of DEMs and 91.04% (1,240/1,362) of DEGs were significantly altered, are inversely correlated and involved in regulatory networking in multiple myeloma. The most downregulated miR observed in MM malignant plasma cells as compared to NPCs in our study is miR-155. A reduced expression of this miR in MM PCs vs NPCs suggests a tumor suppressor role as has also been reported previously^55^. A similar study has reported an epigenetic repression of miR-375 in MGUS and MM primary cells as compared to NPCs^56^, which is also concurrent to our findings. Another tumor suppressor miR-144 that can be sponged by *lncSOX2OT*^57^ has been reported to be downregulated in MM plasma cells and cell lines earlier and was found downregulated in plasma cells in our study. Similarly, upregulation of miR-29b in MM PCs in this study is in sync with previous studies, where it has been reported that the overexpression of miR-29b induces apoptosis of multiple myeloma cells by down regulating *MCL-1*^58^.

Some of the DEMs observed in MM in our study can be extrapolated and categorized on the basis of their previously reported roles relating to pathogenesis, clinical presentation, drug resistance and clinical outcomes. While the deregulated miRs-30d and 181b have been associated with p53 expression^33^, miRs-106/ 181b and miR-181b/ miR-193b are specifically dysregulated in early and late stages of pathogenesis in MGUS and MM respectively^30^. Some of the DEMs have been associated with sensitivities to Bortezomib (e.g., miRs-17-5p, miR-29b-3p, miR-20a-5p) while others with poor survival outcomes (miR-92a, miR-16, let-7e, miR-19b, miR-19a)^25^. Although sample size of inhouse MM subset (n = 44) in our study is small, we observed all the Union37 DEMs in this patient population. Moreover, a significant association of low expression of miR-30d-3p with poor OS and of high expression of miR-16–2-3p with poor OS and PFS (Fig. 4) was also noted. The miR-30d-3p is a known prognostic biomarker for MM reported to have lower serum expression levels and tumor suppressor functions mediated through direct targeting of TP53 and MTDH/PI3K/Akt signaling pathway^59^. A recent study has reported high expression of miR-16–2-3p in serum of Bortezomib refractory MM patients^60^ but its role in MM has not been investigated thoroughly. Since miR-16–2 can target WNT5A, impair ability of MSCs to differentiate into osteoblasts^61^ its deregulation may be of prognostic significance in MM and needs to be explored further. Coincidentally, IPA analysis has also highlighted importance of WNT pathway in this study.

Another integrative study^27^ mined two miRNA and two mRNA microarray GEO datasets and identified 39 DEMs and 32 hub genes. Among these DEMs, miR-155 and miR-148 were found to be deregulated in their study^27^ as well as in Union 37 profile in the present work. Likewise, another meta-analysis of 7 datasets including MM patients^26^ highlighted 13 DEMs, of which hsa-miR-106b, miR-15b, miR-191, miR-19a and miR-20a are also represented in Union 37 profile. A recent meta-analysis by Xu et al^25^ reported 7 DEMs of poor prognostic significance among which deregulated miR-92a, miR-16, let-7e and 19b are common to the Union 37 signature.

The IPA core analysis disclosed 12.42% (n = 154) of DEGs as putative biomarkers that could be useful in diagnosis, disease progression, efficacy, prognosis, response to therapy and safety. Further investigation revealed that 42.21% (n = 65) of targets were involved in more than one functional role. It is known that proteins with the highest degree have the highest betweenness in the network. As hub proteins are accountable for holding networks together^62,63^, they are more likely to be master regulators of signaling and transcription and can be used as therapeutic targets or biomarkers^64^. The target genes identified in this study were subjected to PPI network which disclosed a total of 394 nodes with ≥ 10 connections with other nodes and were designated as ‘hub’ genes. All hub genes were observed to be targeted by MM associated DEMs and could act as possible biomarkers for this disease.

It is noteworthy that IPA based data mining of DEGs and DEMs in this study has revealed five top hub genes lying in the centre of functional networks. These include *UBC, ITGA4, HSP90AB1, VCAM1* and *VCP*. Two genes (*UBC* and *HSP90B1*) have been earlier reported to be upregulated and involved in myelomagenesis in malignant plasma cells in other studies^65^ as well and may be critically involved in ubiqutin-proteosomal pathway. The HSP90A family members are known to promote anti tumor immunity via their exposure on dying myeloma cells^66^ and their interaction with lncRNA *MALAT1* is associated with poor prognosis^67^. Gene *ITGA4* along with *ITGB1* codes for integrin VLA4 that mediates homing of myeloma cells into bone marrow and augment IL6 in the microenvironment.^68^. Similarly, MM cells establish contact with bone marrow stromal cells via adhesion molecules such as *VCAM1* and enhance osteoclast stimulating activity that can be reduced by Bortezomib and Lenalidomide^69,70^. The gene *VCP* is a potential therapeutic target that mediates delivery of ubiquinated misfolded protein aggregates to proteasome^71^ and was found to be upregulated in MM plasma cells in this study.

## Conclusions

The regulatory crosstalk between DEGs and DEMs in MM is highly complex. This study has identified core putative signatures of DEMs (‘Union 37’) and DEGs (‘Union 154’) in MM as compared to normal PCs that may impact clinical outcomes (for instance, miR-16–2 and miR-30d). Further studies on functionally connected hub genes (such as *UBC, ITGA4, HSP90AB1, VCAM1, VCP*), other potential seed genes (e.g., *CDKN1A, CDKN2A, MMP9, IGF1, MKI67*), DEMs and their multidimensional networking with regulatory transcription factors are needed for better understanding of their oncogenic/ anti tumor properties and to explore their synergistic prognostic value.

## Supplementary Information

Supplementary Information 1.

Supplementary Information 2.

## Data Availability

Gene expression (GSE125361) and miRNA expression (GSE125363) signatures in multiple myeloma have been submitted to the National Center for Biotechnology Information (NCBI; https://www.ncbi.nlm.nih.gov/geo) under BioProject accession number PRJNA515992.

## Abbreviations

BP: Biological process
DAVID: Database for annotation, visualization and integrated discovery
DEG: Differentially expressed genes
DEM: Differentially expressed miRNAs
FDR: False discovery rate
GEO: Gene expression omnibus
GO: Gene ontology
IPA: Ingenuity pathway analysis
KEGG: Kyoto encyclopedia of genes and genomes
MM: Multiple myeloma
miRNA: MicroRNA
NCBI: National Center for Biotechnology Information
PPI: Protein–protein interaction
