# Supplementary material for: Genome-wide identification of potential biomarkers in multiple myeloma using meta-analysis of mRNA and miRNA expression data
Source: Sci Rep. 2021 May 26;11:10957. doi: 10.1038/s41598-021-90424-y (PMC8154993; doi:10.1038/s41598-021-90424-y)
Supplement: Supplementary file 2 — Supplementary Information 2. [file 41598_2021_90424_MOESM2_ESM.pptx]

## Slide 1
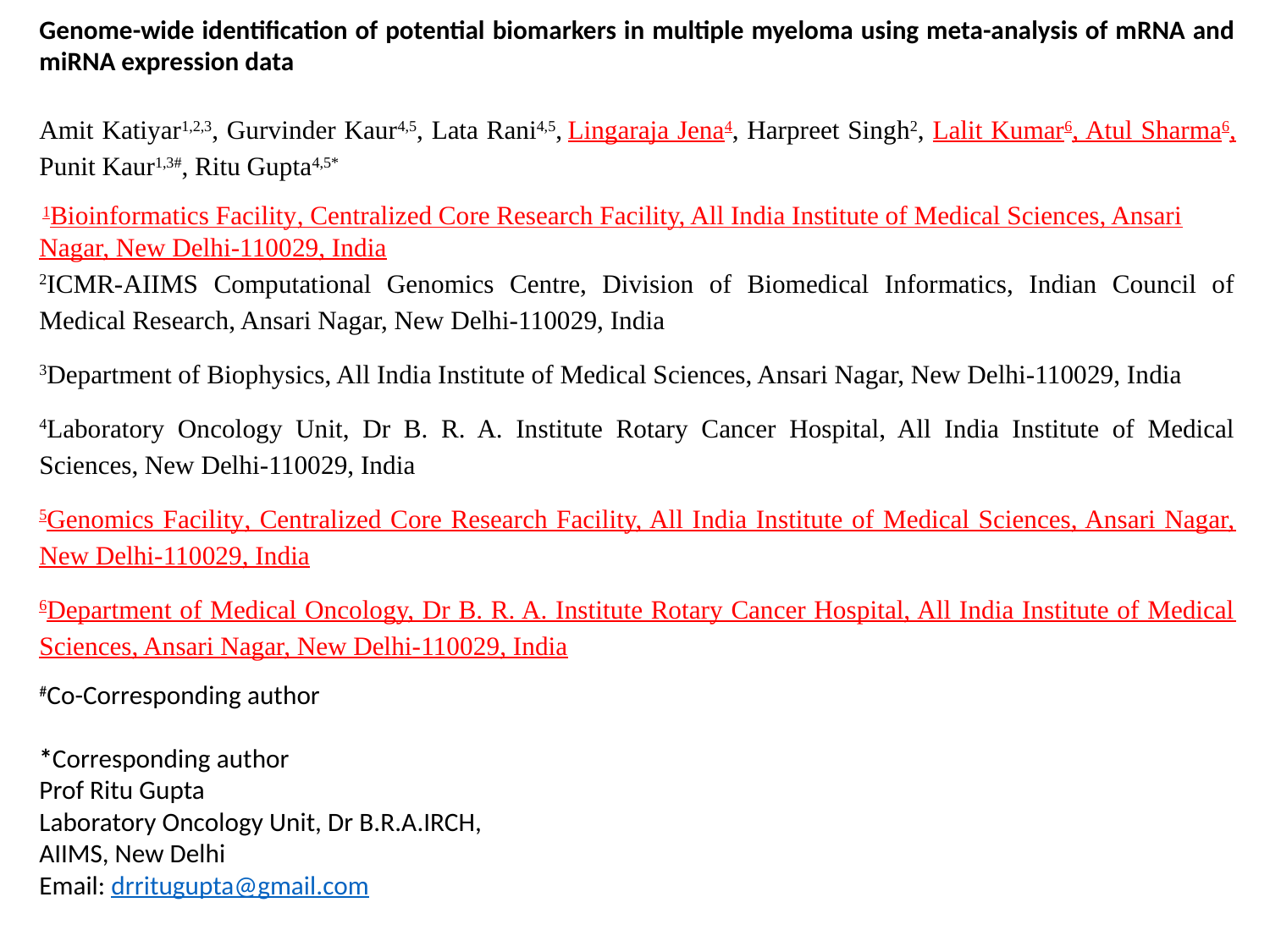

Genome-wide identification of potential biomarkers in multiple myeloma using meta-analysis of mRNA and miRNA expression data
Amit Katiyar1,2,3, Gurvinder Kaur4,5, Lata Rani4,5, Lingaraja Jena4, Harpreet Singh2, Lalit Kumar6, Atul Sharma6, Punit Kaur1,3#, Ritu Gupta4,5*
 1Bioinformatics Facility, Centralized Core Research Facility, All India Institute of Medical Sciences, Ansari Nagar, New Delhi-110029, India
2ICMR-AIIMS Computational Genomics Centre, Division of Biomedical Informatics, Indian Council of Medical Research, Ansari Nagar, New Delhi-110029, India
3Department of Biophysics, All India Institute of Medical Sciences, Ansari Nagar, New Delhi-110029, India
4Laboratory Oncology Unit, Dr B. R. A. Institute Rotary Cancer Hospital, All India Institute of Medical Sciences, New Delhi-110029, India
5Genomics Facility, Centralized Core Research Facility, All India Institute of Medical Sciences, Ansari Nagar, New Delhi-110029, India
6Department of Medical Oncology, Dr B. R. A. Institute Rotary Cancer Hospital, All India Institute of Medical Sciences, Ansari Nagar, New Delhi-110029, India
#Co-Corresponding author
*Corresponding author
Prof Ritu Gupta
Laboratory Oncology Unit, Dr B.R.A.IRCH,
AIIMS, New Delhi
Email: drritugupta@gmail.com

## Slide 2
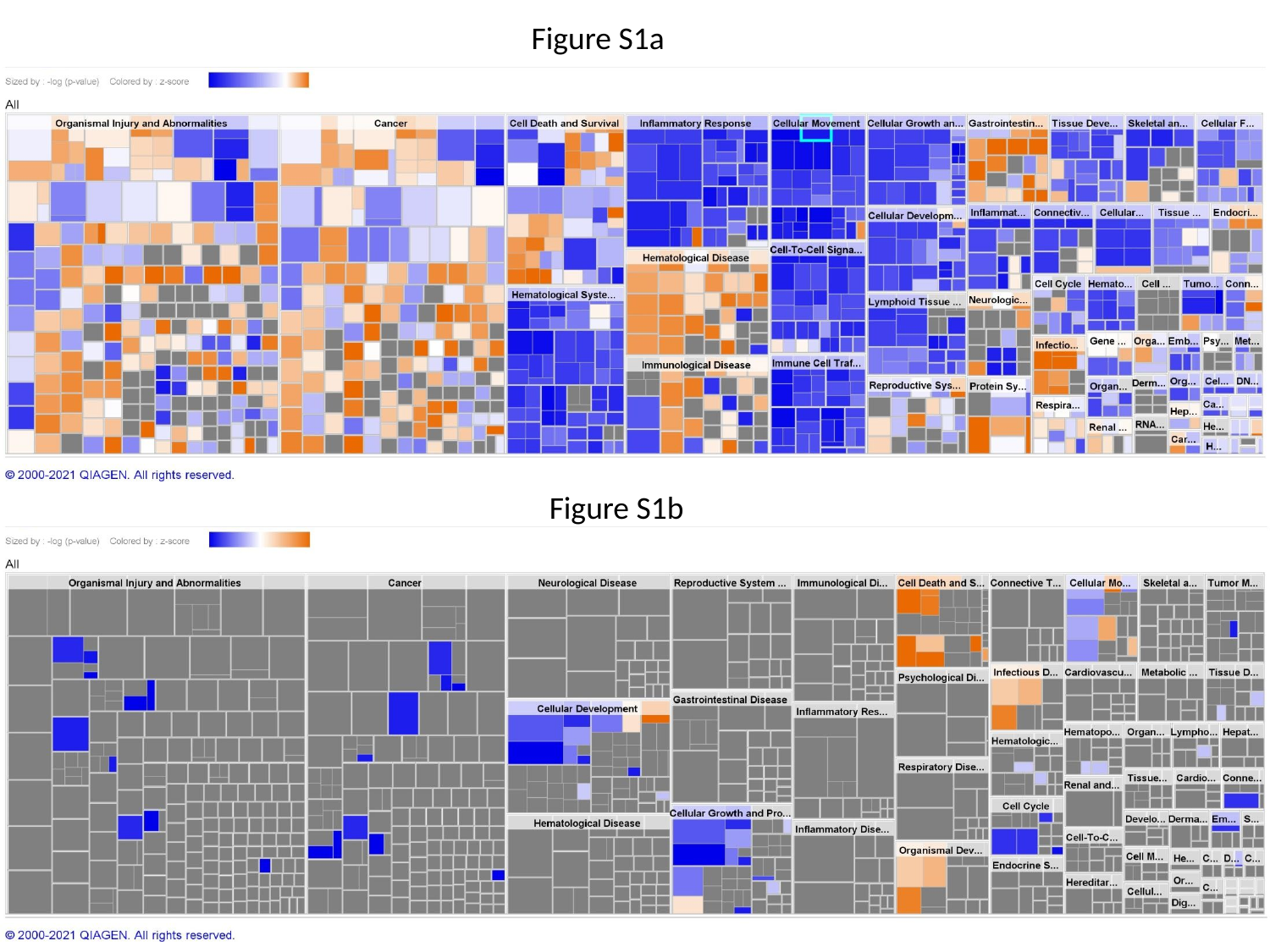

Figure S1a
Figure S1b

## Slide 3
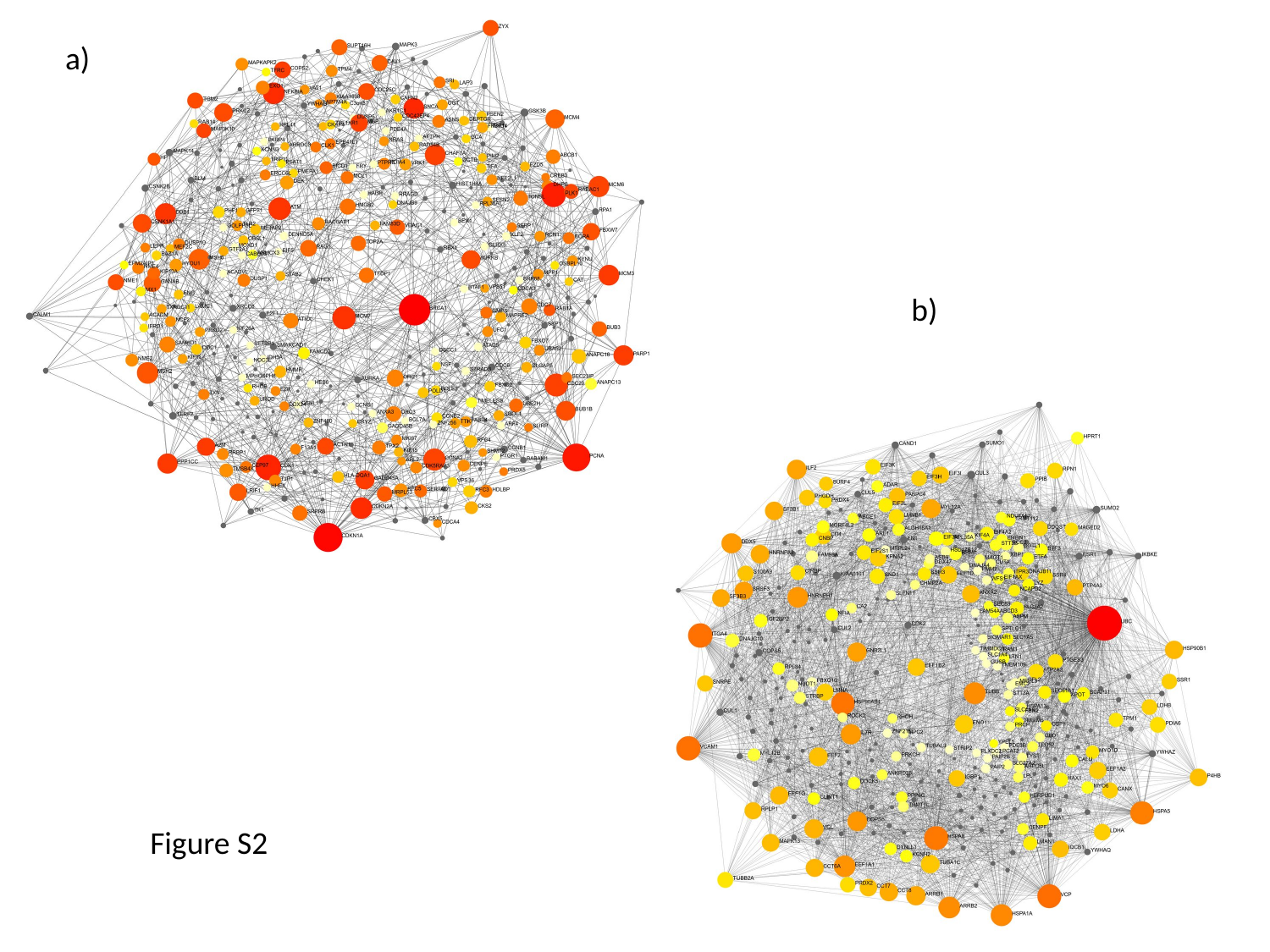

a)
b)
Figure S2

## Slide 4
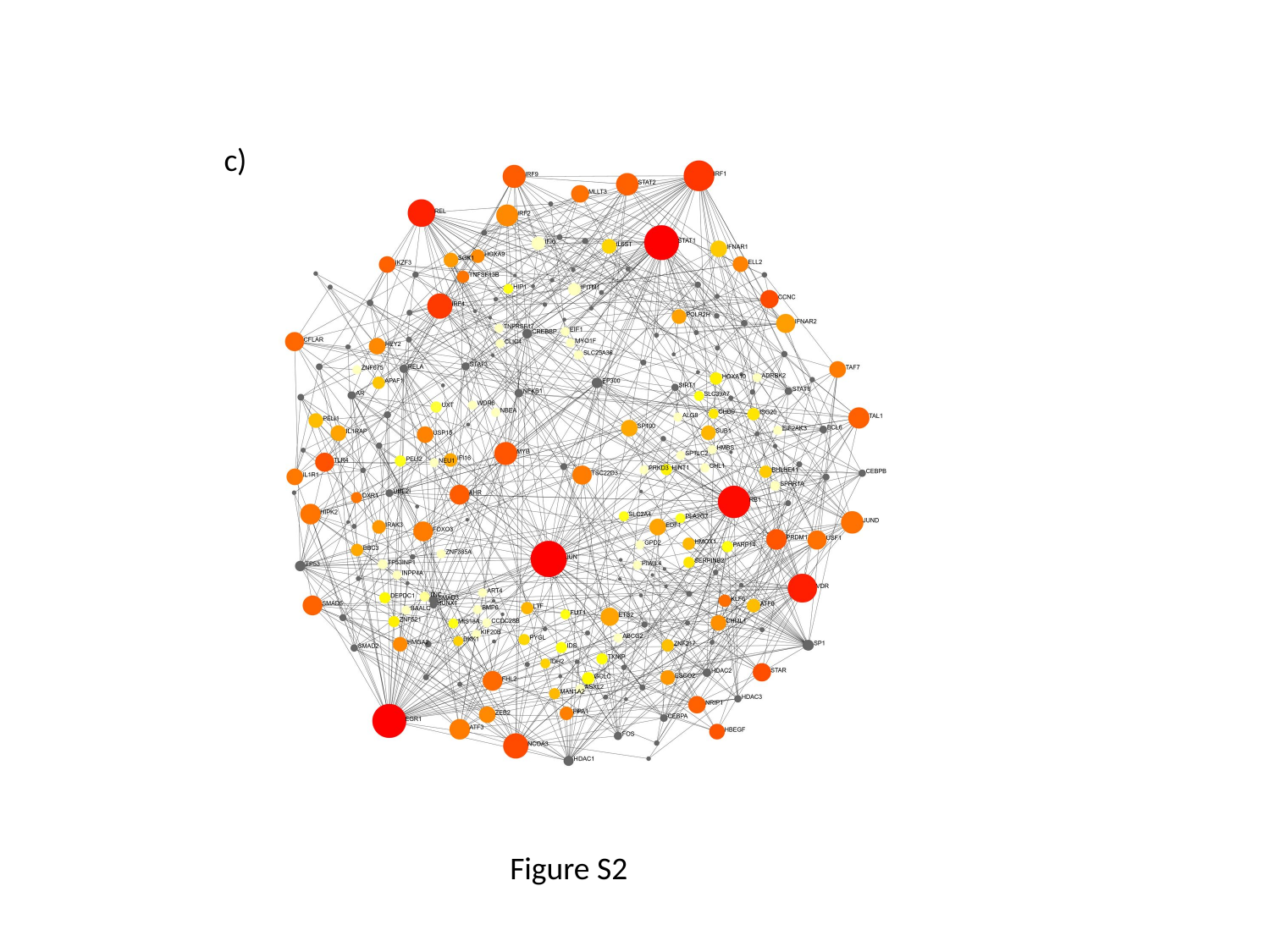

c)
Figure S2

## Slide 5
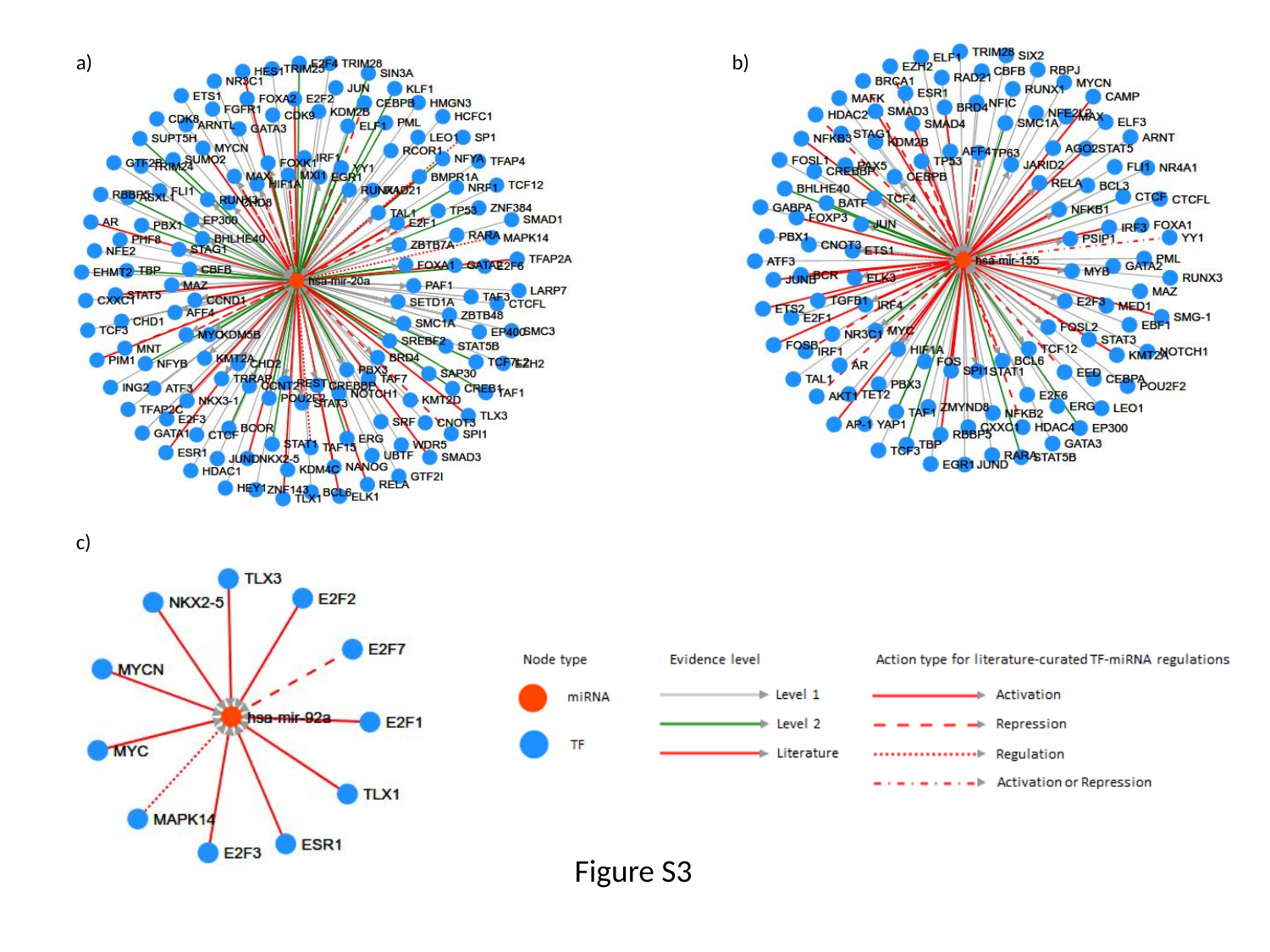

b)
c)
Figure S3
a)

## Slide 6
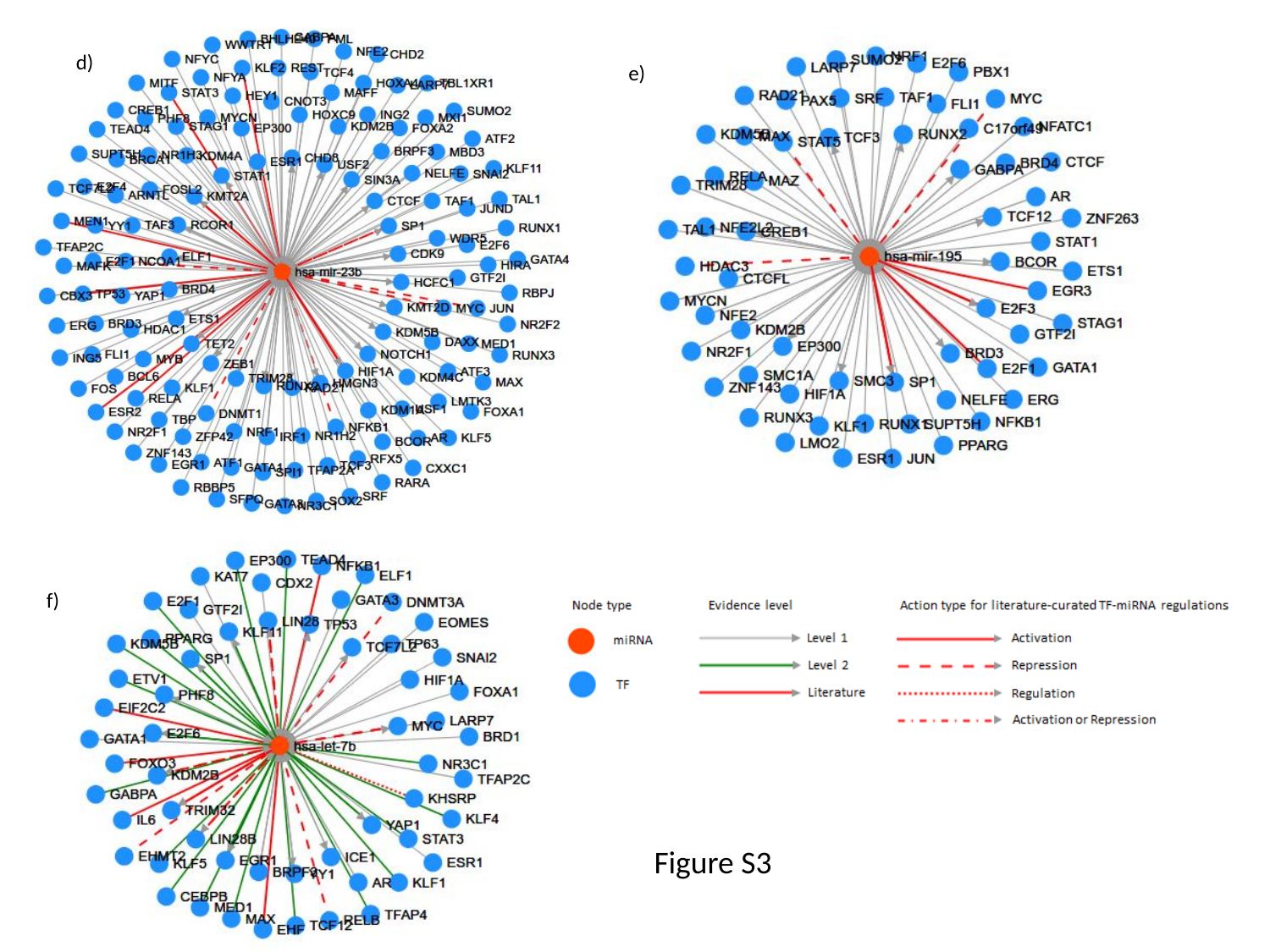

e)
f)
Figure S3
d)
